# The Importance of Lung Innate Immunity During Health and Disease

**DOI:** 10.3390/pathogens14010091

**Published:** 2025-01-17

**Authors:** Gusty Rizky Teguh Ryanto, Ratoe Suraya, Tatsuya Nagano

**Affiliations:** 1 Laboratory of Clinical Pharmaceutical Science, Kobe Pharmaceutical University, Kobe 658-8558, Japan; 2 Division of Respiratory Medicine, Department of Internal Medicine, Kobe University Graduate School of Medicine, Kobe 650-0017, Japan

**Keywords:** lung innate immunity, lung infections, COPD, asthma, pulmonary fibrosis

## Abstract

The lung is a vital organ for the body as the main source of oxygen input. Importantly, it is also an internal organ that has direct contact with the outside world. Innate immunity is a vital protective system in various organs, whereas, in the case of the lung, it helps maintain a healthy, functioning cellular and molecular environment and prevents any overt damage caused by pathogens or other inflammatory processes. Disturbances in lung innate immunity properties and processes, whether over-responsiveness of the process triggered by innate immunity or lack of responses due to dysfunctions in the immune cells that make up the innate immunity system of the lung, could be correlated to various pathological conditions. In this review, we discuss globally how the components of lung innate immunity are important not only for maintaining lung homeostasis but also during the pathophysiology of notable lung diseases beyond acute pulmonary infections, including chronic obstructive pulmonary disease (COPD), asthma, and pulmonary fibrosis.

## 1. Introduction

As an organ continuously exposed to the outside, circulating air, the lung is constantly exposed to various environmental pathogens and toxins. Beyond the obvious risk of microorganism infection, this exposure could also lead to chronic lung diseases with dire consequences [1,2,3]. Central to the pathophysiology of the diseases is the inflammatory process governed by the immune cells of the body [4,5]. Importantly, the innate immune system is the first line of defense against any perceived changes due to various pathological triggers. The lung’s innate immune system, in particular, is highly complex, with multiple cellular components that together orchestrate tissue response to stimuli [6]. Dysfunction in this delicately balanced system has been linked to the development of not only acute pulmonary infections but also various other chronic lung diseases, as reported in numerous studies in recent years [7,8,9]. In this review, we will briefly discuss which cells compose the lung’s innate immunity system, how they can work together in concert during pathological insults, and what happens to them during various diseases.

## 2. Components of Lung Innate Immunity

As with any other organ, the lung has its own set of resident immune cells in addition to the circulating cells that are ready to be called upon to act whenever any pathological stimuli trigger the lung cells [6,10,11]. In this review, we will first focus on the physiological role of the immune cells that make up the innate immunity of the lung, starting with macrophages. As one of the most well-known immune cells, macrophages have been widely studied in the lungs and other organs. The lung has its resident macrophages, divided broadly into alveolar macrophages (AMs) and interstitial macrophages (IM) [5,12]. AMs found adjacent to the alveolar airspace are considered cells that are functionally adaptable to their microenvironment and differentiation states [13]. AMs are traditionally thought of as anti-inflammatory, although recent evidence suggests that this could be more complex [14]. Their major role is performing phagocytosis of particulate matter, dying cells, and cellular debris, as their continuous exposure to environmental stimuli would suggest [14,15]. This is important in limiting lung inflammation to avoid excessive inflammatory responses to external stimuli, and, as such, under homeostatic conditions, AMs are largely kept in a quiescent state [15]. AMs are mostly self-renewing and do not rely on bone marrow for fresh populations [16]. Most AMs originate separately from the circulating, common monocytes and/or precursor cells. A small subset of fresh AMs can be recruited from the circulation, however, and over time gain the characteristics of AMs already residing in the lung tissue [17]. AMs are important not only in phagocytosis but also in maintaining the homeostasis of other cells in the tissue, including the epithelial cells, dendritic cells (DC), and T-cells, among others. This is done by producing several molecules such as TGF-*β*, interleukin-6 (IL-6), and RANTES, among other molecules [18].

The origin of lung interstitial macrophages (IM) that reside in the lung parenchyma, on the other hand, is not completely known. It was previously thought that IMs are macrophages that are in the interim state between those recruited from the circulation and the resident AMs, but recent studies have shown how IMs are transcriptionally, ontogenically, and functionally different from AMs [19]. First, differing from AMs, IMs seem to rely on the circulating cells to replenish themselves [11,19]. What complicates the matter is that reports have suggested that there are multiple IMs with different functions and locations. For example, peribronchial IMs express CD206 and MHCII and function during the immunoregulation and wound-healing process. IMs without CD206 expression and low CX3CR31 expression are instead involved in antigen presentation and interact with the interstitium of the alveoli, in addition to being continually replenished by the circulating monocytes, while another population is detected perivascularly [20]. One of the ways IMs play a key role in immunoregulation is by secreting immunoregulatory cytokines such as IL-10 [21]. Furthermore, they are capable of performing small particle phagocytosis (to a lesser degree than AMs) and chemotaxis and have the ability to produce reactive oxygen species. Lastly, the antigen-presenting capacity of IMs is superior to that of AMs and promotes T-cell proliferation and Treg cell differentiation [22].

Beyond macrophages, other types of immune cells are also in play in lung innate immunity system. For instance, while neutrophils were previously thought of as recruited from the circulation, recent studies highlighted how neutrophils can reside in the lung tissue even during normal conditions and regulate the status quo [23]. Compared to those circulating, lung neutrophils differ in phenotypes and specific functions, with one report mentioning its high production of interleukin (IL)-6 and low levels of tumor necrosis factor-*α* (TNF-*α*) after stimulation [23]. In the lung, neutrophils are involved in the removal of cellular debris while also being a major trigger of the acute lung inflammation process, which can be pathogenic because it promotes further tissue damage [24]. In the later stage, neutrophilic infiltration performs its tasks in the damaged parts of the tissue to promote regeneration, which depending on the situation could be beneficial or damaging. This process is mediated by, among others, neutrophil extracellular traps (NETs), matrix metalloproteinase (MMP)-2, MMP-9, lipoxins, resolvins, and protectins [5,25].

Innate lymphoid cells (ILCs) are a diverse group of lymphoid cells resident in the peripheral tissue (in this case the lung) that have similarities to T-cells, only without the antigen-specific receptors [26]. They instead respond to locally secreted cytokines by other components of innate immunity in the lung. The three ILC subtypes, named simply as ILC1, ILC2, and ILC3, are, respectively, similar to T-helper (Th) 1, T-helper 2, and T-helper 17 cells [11,26]. Much like other resident immune cells, ILCs can also change their phenotype and function to adapt to their environment, while being able to self-renew in various settings [27]. Using the most abundant ILC subtypes of ILC2 as an example, one study showed how in the two months after birth, only 5–10% of lung-resident ILC2s were of embryonic origin, showing how ILCs can renew themselves de novo [28]. ILC1 expresses residency markers such as CD69 and CD103 and is relatively low in number in the lung, but these cells are important to survey and control for any possible infection in the lung [29]. They secrete interferon (IFN)-*γ* in response to interleukins 12, 15, and 18 to boost other immune cells to eliminate potential pathogens [26,29]. ILC2, on the other hand, is involved in the production of type 2 cytokines IL-4, IL-5, and IL-13 upon stimulation by IL-25 or IL-33, among others [30]. These cytokines are known as important mediators in allergic responses and during asthma [30]. ILC2s comprise the main population of ILCs in the lung, and they express the surface markers IL-7Ra, CD25, ST2, and CD44, among others [26]. Lastly, ILC3 expresses the retinoic acid receptor-related orphan receptor *γ*t transcription factor (ROR*γ*t) and is thought of as similar to T-helper 17/22 cells [31]. As the analogy suggests, ILC-3 secrete IL-17 and IL-22, both of which are key mediators in lung immunity [11,32]. IL-18 and GATA3 are known to promote ILC-3 maintenance, proliferation, and cytokine production [32].

Natural killer (NK) cells are part of the ILC-1 family due to similarities in transcription factor requirements and IFN-*γ*production, but they function as cytolytic cells instead and can degranulate upon stimulation by infections [33]. The lung contains several distinct populations of NK cells, which are mainly based on the expression levels of CD56, CD16, and NKp46 [34]. The lung-resident NK cells have diminished CD56 levels with positive CD16 phenotype and are negative for CD69 [34]. They have already differentiated and do not elicit a high level of response to target cell stimulation [34]. Another study reported that lung-resident NK cells are those with the expression of CD49a, CD69, and CD103, which are more suggestive of tissue residency than the previous population might suggest [35]. Dendritic cells (DCs) are antigen-presenting cells that, in the lung, work to process inhaled pathogens and migrate to lymph nodes [36]. There, they present the processed pathogen peptides to T-cells [37]. While DCs mostly need to be replaced by fresh ones deriving from the monocytes and the bone marrow, there are populations of DC precursors found in the lung. The three known subsets of DCs are the two conventional DCs (cDCs), aptly named cDC1 and cDC2, and the plasmacytoid DCs [38]. cDC-1 and cDC-2 are distinguished by the expression of CD103 (positive in cDC1, negative in cDC2) and CD11b (negative in cDC1, positive in cDC2) [5,39]. cDC-1s are adjacent to the airway epithelium while cDC2s are mostly found in the lung interstitium, much like pDCs [5].

Finally, beyond these cell types, mast cells (MCs), basophils, and eosinophils are also important innate immunity responders, especially during allergic inflammation [40]. MCs originate from the bone marrow and reside in lung tissues to survive for months. During an allergy, once an individual has been exposed to enough of the allergen to develop the antigen-specific IgE that is bound to FcεRI (the high-affinity IgE receptor), allergen re-exposure will cause the crosslinking and aggregation of the neighboring FcεRI-bound IgE [41]. This will trigger MCs to immediately release mediators of the allergic reactions, including histamine, serotonin, prostaglandin, leukotriene, and protease [42]. MCs can also release inflammatory cytokines and chemokines after activation [42]. Another cell type similar to MCs is the basophils. They also express the high-affinity receptor FcεRI and can release similar mediators such as histamine and other cytokines, although basophils have a relatively shorter lifespan than MCs [43]. T-cell-derived IL-33 is known to promote basophil development [44]. Lastly, the lesser-known eosinophils have been associated with the pathogenesis of asthma and they are known to be accumulated during allergic asthma to mediate efferocytosis and apoptosis, but the molecular details regarding their role in the lung remain to be elucidated [45]. Together, these different immune cell types not only orchestrate the lung response to external stimuli but also preserve the necessary molecular balance needed for the lung cells to maintain themselves. Disturbance in this delicate system, as will be discussed later, is detrimental to limiting the pathogenic process in various acute and/or chronic inflammatory diseases in the lung. On the other hand, pathological conditions can also drive these immune cells to dysfunction. We will further discuss this phenomenon in the context of each pulmonary disease.

## 3. The Role of Innate Immunity During Lung Diseases

### 3.1. Lung Infections

Infections by pathogenic microorganisms are the most obvious condition in which the lung’s innate immunity system plays its role [8]. As briefly touched on in the previous section, multiple layers of processes involving most, if not all, of the innate immune cellular component are in play to limit the infection, whether viral, bacterial, or helminthic [11]. Moreover, any functional or molecular changes due to genetic changes or variations in the innate immune cells could also contribute to the difference in how the immune system responds to infections.

Starting with the macrophages, the role of AMs during infections is to induce effective defense mechanisms against said pathogens. Studies have shown that when, among others, *Mycobacterium tuberculosis*, *Streptococcus pneumoniae*, and *L. pneumophila* infect the lung, they will activate AMs to produce cytokines and chemokines such as IL-1*α*, IL-1*β*, IL-6, TNF-*α*, type 1 interferon (IFN-*α*/IFN-*β*), TGF-*β*, and prostaglandin-E2 [10,14]. In addition, the expression of macrophage receptors with collagenous structure (MARCO) in AMs helps them in phagocytic clearing of said pathogens, and expressional reduction or mutation in the MARCO gene is associated with reduced AM phagocytosis capacity and increased inflammation [46]. H101Q heterozygous variation in the MARCO gene is also associated with sepsis from lung infections [47]. Genetic variations in genes encoding the cytokines, such as the IL-6, IL-1*α*, IL-1*β,* or the interferon type 1 gene, have also been related to increased severity of various viral infections, such as RSV or influenza [48]. Furthermore, the phagocytosis process of apoptotic cells by AMs can also prevent intracellular contents that might be inflammatory and induce additional damage to surrounding tissue [49]. AMs are also known to release small, cationic anti-microbial peptides such as beta-defensins [50]. In humans, beta-defensin 2 is most abundantly expressed in the lung and reacts to specific components in both gram-negative and gram-positive bacteria as its attractant [51]. Additionally, beta-defensin 2 is reported to be effective against various microbiomes, such as *Staphylococcus aureus*, *E. coli*, and *Klebsiella pneumoniae*, among others [52]. Lastly, beta-defensins can also act as an immune enhancer and chemotactic factor for other immune cells [52].

In comparison, the role of IMs is less known, but several studies have suggested that it could work similarly to AMs in different pathogens. Recent studies have highlighted its importance in various viral and bacterial infections, including after SARS-CoV-2 infection [53]. SARS-CoV-2 was shown to predominantly infect activated interstitial macrophages (IMs) using the cell transcriptomic capability to form RNA bodies and eliciting pro-fibrotic and inflammatory cytokine release from the host, such as IL-6, CXCL (C-X-C motif chemokine ligand)-10, SPP1, and TGF-*β*, among others [53]. Others have reported how IMs and not AMs mediate the efferocytosis of alveolar type II epithelial cells (AT2 cells) influenza infection [54]. Notably, the BCG (bacille Calmette–Guérin) vaccination could boost the nonspecific protective effects of monocyte-derived immune cells from various non-tuberculosis microbial infections through the increase in IFN-*γ*, TNF-*α*, and IL-1*β* productions via a NOD-2-dependent pathway [55]. Not only in bacterial infections, BCG vaccinations also offer similar non-specific enhancement in macrophages, either monocyte-derived or the resident AMs, responses after viral infections, either via direct enhancement of cytokine productions or via a gut–lung axis that modulates the intestinal microbiome, which affects the circulating lung metabolites [56,57]. Nonetheless, the role of IMs and how it differs from AMs needs to be explored more in future studies.

During infections, the role of neutrophils is to help trigger the acute inflammatory response and trigger the removal of endogenous and exogenous debris [58]. Due to their nature, neutrophils can be viewed as pathogenic because when activated, they will cause more damage in the early stages of inflammation, which is attributable to the release of the pro-inflammatory cytokines and chemokines such as TNF-*α*, IFN-*γ*, IL-8, CCL (C-C motif chemokine ligand)-2, and CCL-7 [59]. Other peptides released by neutrophils include the neutrophil peptides cathelicidin LL-37 and lipocalin 2, both of which are microbicidal [60]. Significantly, LL-37 is also important to various immunomodulating mechanisms beyond the anti-microbial activity, including the stabilization of NETs against bacterial nucleases, supporting the differentiation of Th17, or work as a chemoattractant for other immune cells, among other functions [61,62]. The trigger to the acute inflammatory response by neutrophils is the binding of the antigen or various pro-inflammatory cytokines or peptides (e.g., lipocalin-2) to specific receptors such as the toll-like receptors (TLR) family, which will start the release of multiple mediators and induce the recruitment of neutrophils to the injury site, which in the case of the lung is the alveolar space [24,59,63]. For example, *P. aeruginosa*, lipopolysaccharide (LPS), and *β*-glucans can induce the recruitment of neutrophils to trigger the acute inflammatory process [24]. Importantly, genetic variations in single nucleotide polymorphisms (SNPs) in some of the TLR (e.g., TLR2, TLR3, TLR4, and TLR8) genes are reported to alter the severity of infections such as RSV [48,64]. SNP in the IL-8 encoding gene is also associated with more severe symptoms of RSV [65].

In the case of ILCs, several lines of evidence point to the diversity of the role this cell has depending on its subtype. For example, ILC1s can be activated and secrete IFN-*γ* and TNF-*α* after infection with the H1N1 influenza virus as early as three days after infection [26,66]. In line with that result, another study showed how ILC1 depletion in T-cell deficient mice caused a titer increase of Sendai virus in the lung after infection [29]. ILC2s, on the other hand, receive signals from infected epithelial cells and can swiftly release several cytokines, including the aforementioned IL-4, IL-5, and IL-13, in addition to TGF-*β* and amphiregulin, among other cytokines [67]. Lastly, ILC3s are vital in lung infections and also due to their capability to produce IL-17 and IL-22 [68]. Both of these molecules are important in the clearance and protection from bacterial and viral infections, such as *S. pneumoniae* and *M. tuberculosis* [68,69]. Further, the reduction in epithelial regeneration capability in influenza-infected IL-22 knockout mice could be restored by transferring ILC3 cells into the mice or treating it with recombinant IL-22 [26]. Again, genetic variations in the form of SNPs in the IL-4 and/or IL-13-encoding gene have been correlated with increased RSV severity [48,70,71].

**Figure 1:**
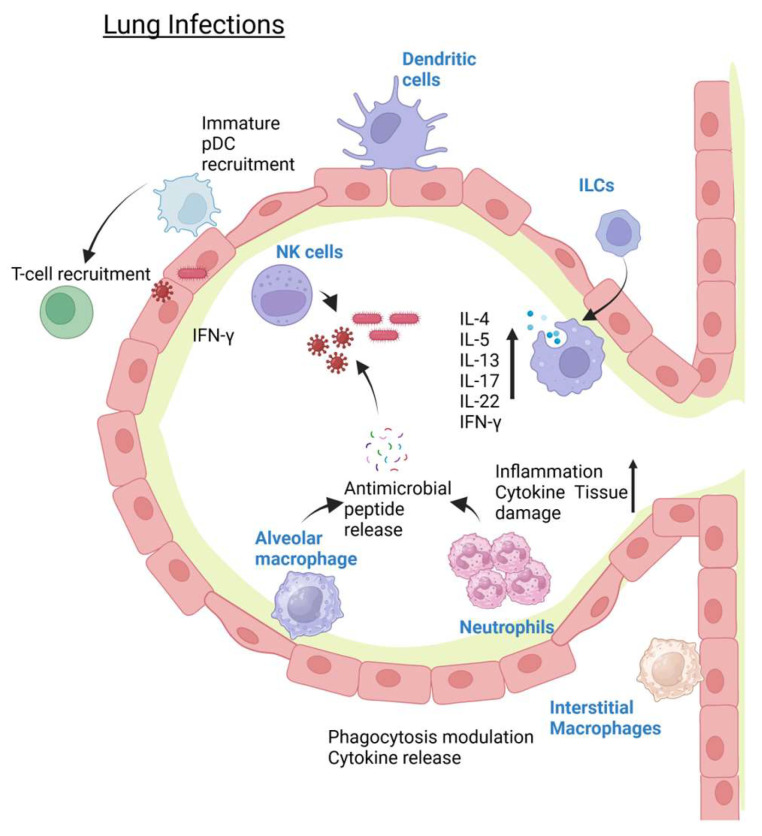
Changes seen in the innate immunity component during lung infections. Created in Biorender. Nagano, T. (2025). https://BioRender.com/a87c229 (accessed on 15 January 2025).

Another producer of the vital cytokine IFN-*γ* in the lung during infection is the NK cells. As they are one of the first lines of defense against pathogens, the ability to secrete a protective cytokine such as IFN-*γ* is vital in limiting disease severity [72]. Variations in the gene encoding IFN-*γ* (IFNG) through SNPs are more frequently found in patients with pulmonary infections, such as those with COVID-19 infections [73]. NK cells can also produce IL-21 and IL-22 to enhance local immune responses by other immune cells, such as the circulating monocytes or the resident macrophages [74]. True to their name, NK cells are also important in pathogen clearance in harmony with macrophages, and also in the direct killing of pathogens [75]. In the case of DCs, as their main role is to process the pathogens and introduce them to T-cells, DC subsets contribute to the control of microorganism infection burden [76]. Some studies have indicated how the number of pDCs increased following infections [77]. One example is the increased number of pDCs in the lung after *Klebsiella pneumonia* infection, which subsequently corresponds with increased antigen-specific CD4+ T-cell responses [78]. Another example is how *Pasteurella multocida* infection can trigger DC maturation and IL-12 production that can induce naïve T-cell maturation [79]. The changes in innate immunity during lung infections are shown in Figure 1.

### 3.2. COPD

In the case of chronic lung diseases such as COPD, the innate immune cells are important not only during acute exacerbations due to infections but also contribute greatly to the adverse airway remodeling that can be seen in the lung. During COPD progression, damage-associated molecular patterns (DAMP) could be triggered by the mixture of pathogen infiltration and dissolution, impaired immune cell functions, microenvironmental changes, and any other insult or injury to the airway [80]. Triggering DAMPs will result in the pattern recognition receptors (PRR), such as the aforementioned TLRs, nucleotide-binding oligomerization domain receptors (NOD-like receptors or NLRs), C-type lectin receptors, retinoic acid-inducible gene 1(RIG-1)-like receptors (RLRs) and cytosolic DNA receptors, to recruit and activate the innate immune cells [81,82]. During COPD, immune cell PRRs, such as those in the neutrophils, are overexpressed, and as a result, the number of recruited, activated immune cells increases, which becomes an important prognostic factor for COPD progression and severity [80]. For instance, neutrophil count and chemoattractant levels are regarded as markers of COPD progression and exacerbation [83]. Another example is how the total number of macrophages is also increased in COPD patients [84]. These macrophages are large in size, produce lower levels of pro-inflammatory cytokines (e.g., TNF-*α*, IL-1*β*, and IL-6), and are less capable of phagocytosis [85]. One study highlighted how AM populations in the COPD lung exhibited reduced phagocytic capacity, and how this correlates with impairment in pathogen clearance ability and reduced FEV1% [86]. A subset of the macrophages found in COPD patients exhibit continuous pro-inflammatory effects instead; this is shown by its capability to produce higher levels of pro-inflammatory cytokines and MMPs while enhancing extracellular matrix deposition in the airway and lung, thereby contributing greatly to the airway remodeling seen in COPD [87]. This alternatively activated macrophage phenotype is also true for IMs; one study highlighted how IMs in the peribronchial area of COPD patients are positive for iNOS, arginase I, and YMV [88].

**Figure 2:**
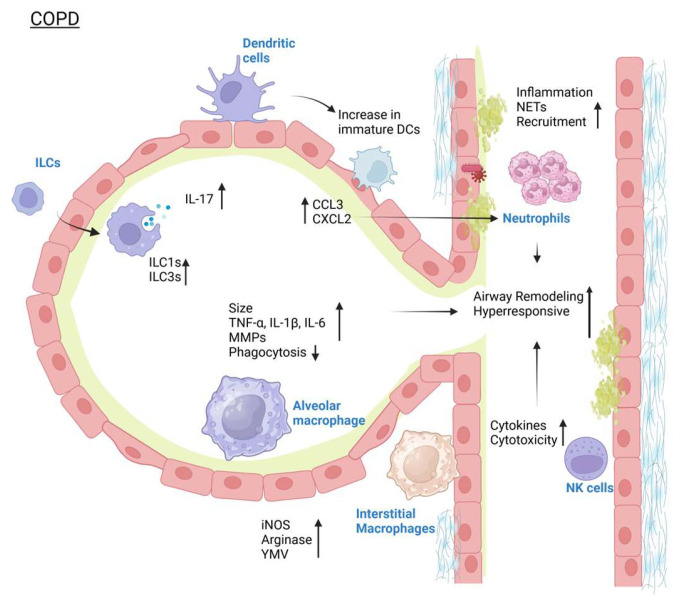
Changes seen in the innate immunity component during COPD. Created in Biorender. Nagano, T. (2025). https://BioRender.com/s24o223 (accessed on 15 January 2025).

The plasticity of ILCs is clearly shown during COPD, where the abundant ILC2 can transition in mass numbers into an ILC1-like state by the molecules IL-12 and IL-18, or by cigarette smoke and bacterial infections [89]. Clinically, this increase in ILC1-like cells is correlated with reduced lung function and disease severity, indicating the potential role of ILC1-like cells in COPD pathology [90]. IL-17 derived from ILC3 is instead needed for survival from infections that commonly occur in COPD, such as *Pseudomonas aeruginosa* [91]. NK cells may also contribute to the chronic inflammatory state found in COPD through their production of pro-inflammatory cytokines and increased cytotoxic capability [92]. NK cells are increased not only in the tissue but also in the sputum and bronchoalveolar fluid lavage of COPD patients [93]. Further, NK cells isolated from COPD patient airways were found to be highly cytotoxic to the lung epithelial cells compared to those that were isolated from the blood, which is mediated by IL-15 through the communications of NK cells with DCs [94]. This could also be attributed to the possibility that NK cells in the lungs of COPD patients are already activated, marked by the increase in granzyme B and perforin expression [95]. In the clinical setting, the increased presence of NK cells is inversely correlated with FEV1% and FEV1/FVC, showing how NK cells could affect the chronic progression of COPD [96]. Lastly, because DCs are important as antigen presenters, they increase in number during COPD, where long-term reduced protection and continuous exposure to harmful pathogens and particles happen [97]. However, this pathogenic condition also impairs DC maturation, thereby limiting its actual role as an antigen-presenting cell. Instead, immature DCs accumulate in the airways of COPD patients in a larger number than normal, and these immature DCs secrete CCL3 and CXCL2, both of which promote neutrophil recruitment to the site [98]. Clinically, this is proven by the correlation between immature DC numbers and FEV1 value, indicating how immature DCs also play a role in COPD progression [98]. The changes in innate immunity during COPD are shown in Figure 2.

### 3.3. Asthma

The correlation between asthma development and innate immunity system dysfunction has been reported in various studies over the years. Early genomic studies indicated that polymorphism in several genes related to PRRs, such as TLRs or NODs, is related to asthma, while a larger genome-wide association study (GWAS) additionally identified IL-33, ST2, and TSLP (thymic stromal lymphoprotein) as being important in asthma [99]. These are some of the genes expressing proteins related to the innate immunity system and underline its connection with asthma. Recent evidence suggests neutrophilic inflammation can be found in one out of five asthmatic lungs, a condition also termed neutrophilic asthma [100]. What confuses matters is that in the particular subset of patients with higher neutrophils, glucocorticoid usage is associated with prolonged neutrophil survival and subsequently persistent increase during asthma [100]. While the role of neutrophils in asthma is unclear, the increased presence and activity of neutrophils in asthma have been related to the presence of bacterial or viral infections which leads to the release of neutrophil elastase and subsequently NETs [101]. NETs, including extracellular DNA (eDNA), and a high number of eDNA in sputum have been associated with poorer asthma control and mucus hypersecretion in patients [102]. Further, TSLP-TLR3 signaling could also trigger naïve T-cell conversion to Th-17 cells, which will recruit more neutrophils to the site of injury [103].

Macrophages are another type of immune cell with a surprisingly high correlation with asthma. In the case of AMs, it is natural to think that AMs could have a protective role in asthma development. However, asthmatic AMs have been shown to differ greatly in functions compared to non-asthmatic AMs concerning their role. While asthmatic AMs produce a greater number of anti-inflammatory IL-10, which corticosteroids can amplify, asthmatic AMs can also produce pro-inflammatory effects that drive the progression of asthma [104]. For instance, AMs are among the facilitators of neutrophil recruitment to the airspace, while allergen-sensitized AMs can also induce eosinophilic inflammation in otherwise healthy lungs of mice [105]. IMs are another source of IL-10, and as with the IL-10 secreted by AMs, they are also important in alleviating asthma development. IL-10 in asthma works by limiting Th2 allergic inflammation and neutrophilic inflammation [21]. IMs can also separately suppress neutrophil NETosis and inflammation through reduced Th17 activation [106].

ILCs, through their function in expressing interleukins, also play a role in asthma pathology. ILC2s in the lung can control eosinophil accumulation, activation, and survival through IL-13 secretion, which is key in allergic asthma [26,107]. In an allergic asthma mouse model, an increased ILC2 number could be observed, which leads to increased IL-5 and IL-13 and worsens allergic inflammation and airway hyperreactivity [107]. This increase in ILC2s, IL-5, and IL-13 can also be observed in the sputum of asthmatic patients [108]. ILC2s can also respond to IL-33 stimulation and produce inflammatory cytokines [44]. ILC3s can also induce inflammatory responses in asthma. ILC3s secreting IL-17 have been reported to induce airway hyperresponsiveness in allergic asthma and obesity-related asthma [31,109]. Further, ILC3s are also increased in the sputum and bronchoalveolar lavage of asthmatic patients, while ILC3s signature genes are also highly expressed in human asthmatic patient samples [110].

In asthmatic patients, NK cells are more cytolytic with higher levels of the cytolytic protein granzyme A [111]. NK cells can also cause allergic sensitization, type-2 immune response, and airway hyperresponsiveness [112]. The activation of NK cells can also attenuate eosinophilic inflammation [113]. In asthma, NK cells are highly activated when there are coinciding bacterial or viral infections and augment the exacerbation reaction, although other reports have also stated how they can prevent further inflammatory reactions to infections [113]. DC populations, such as cDC2 that express CD11b, are also an important part of asthma pathogenesis, as they are the population that introduces the allergens to the T-cells and generates robust Th2 and Th17 after an allergen challenge [39]. The pDCs are another DC subtype that can contribute to the immunosuppression of allergen response by upregulating PD-L1 in the T-cells [114]. However, other studies have stated that pDCs are also able to potentiate Th2 response, much like cDCs, and accelerate allergen-induced asthma [115].

**Figure 3:**
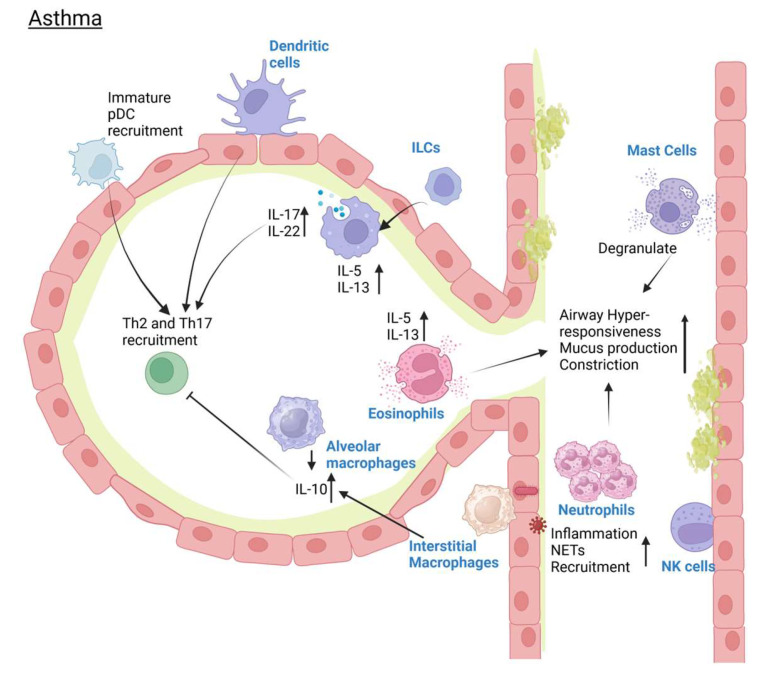
Changes seen in the innate immunity component during asthma. Created in BioRender. Nagano, T. (2025) https://BioRender.com/z34r987 (accessed on 15 January 2025).

Asthma is identical to the increase in MCs numbers, which has been proven correct when looking at the airway of asthmatic patients, even when they are only mildly asthmatic [116]. This is especially true in patients with IL-13 gene signature in their epithelium, where MCs could be easily found and are correlated with high levels of Th2 [117]. MCs will degranulate to a higher degree during fatal asthma, and this degranulation contributes greatly to the augmented response of the airway that leads to exacerbation. The role of eosinophils, meanwhile, has only recently been elucidated in asthma, so much so that there is a specific subset of asthma highlighted by eosinophilic inflammation (termed eosinophilic asthma) [118,119,120]. First, they produce IL-5, and IL-5 is found in asthmatic patients’ bronchoalveolar lavage fluids [120,121]. Recently, many have reported that IL-5 deletion leads to airway eosinophilia because of IL-5’s ability to control the eosinophil recruitment, maturation, activation, and inhibition of apoptosis [119,121]. This is especially important for the eosinophilic asthma subtype, and several IL-5 targeting drugs are currently being studied for use in severe asthma and eosinophilic asthma patients [119,122]. Beyond IL-5, several other factors are known to be able to mediate eosinophil activation, including the crosslinking of Fc*α*RI and Fc*γ*RII with IgA and IgG, the integrin VLA-4 binding to VCAM-1 (vascular cell adhesion molecule-1), IL-25, and IL-33, among others [123]. Eosinophils in asthma contribute to the occurrence of airway hyperresponsiveness, tissue damage, and airway remodeling through their secreted factors, including TGF-*β*, and IL-13, leukotrienes, and eosinophil peroxidase (EPO), while also triggering mast cell degranulation via major basic protein and EPO [123]. Still, more studies are warranted to confirm the role of eosinophils in asthma in the future. Figure 3 summarized the changes seen in innate immunity during asthma.

### 3.4. Pulmonary Fibrosis

Lung fibrosis is another pathological condition where the innate immune system plays a role, and the various components of innate immune cells have been implicated in its pathogenesis. Again, DAMPs and PRRs are central to the promotion of fibrotic remodeling of the lung, where the release of the DAMPs caused by pro-fibrotic triggers leads to the activation of immune cells and subsequent release of cytokines and inflammasome [124,125]. Not only as the trigger, this inflammasome could also be attributed to the progression of fibrosis, where continuous activation of the inflammasome by the stiffened lung and continued mechanosensing by the cells promote an uninterrupted fibrotic process [124,126,127]. This fibrotic process is promoted by the various innate immune cells residing in the lung and recruited from the circulation.

Neutrophils in the bronchoalveolar lavage fluid of idiopathic pulmonary fibrosis (IPF) patients correlate with poorer prognosis and worse clinical outcomes [128]. This neutrophil accumulation is attributable to the increase in IL-8 secreted by the colony-forming cells [129]. Augmented neutrophil degranulation and release of the neutrophil elastase are also related to fibrogenesis, where mice deficient in neutrophil elastase have reduced fibrosis levels [130]. NETosis is another mechanism by which neutrophils could contribute to fibrogenesis, where NETs could induce damage to the lung tissue and force fibrotic remodeling [131]. Besides neutrophils, macrophages have been extensively studied in their relation to pulmonary fibrogenesis. AMs have been strongly related to fibrogenesis in the lung in recent years, and several studies highlighted how they can be alternatively activated by arginase 1, among others, and drive fibrogenesis [132]. AMs from IPF patients are more readily able to secrete pro-inflammatory cytokines and their ability to crosstalk with lung fibroblasts and control ECM production is reduced, thereby promoting fibrogenesis [133]. There is also a subset of SiglecF-positive AMs that is initially lost after bleomycin-induced pulmonary fibrosis induction in mice, while later an increase in SiglecF-low AMs numbers can be seen during the fibrotic phase [17]. This expansion of the AM population in the latter stages of fibrogenesis can also be seen in human lung samples and single-cell RNA sequencing of patient tissues [134]. IMs derived from migrated monocytes also appear to be important in fibrogenesis, where depletion of IMs that express repair-associated genes can promote increased fibrosis [135].

**Figure 4:**
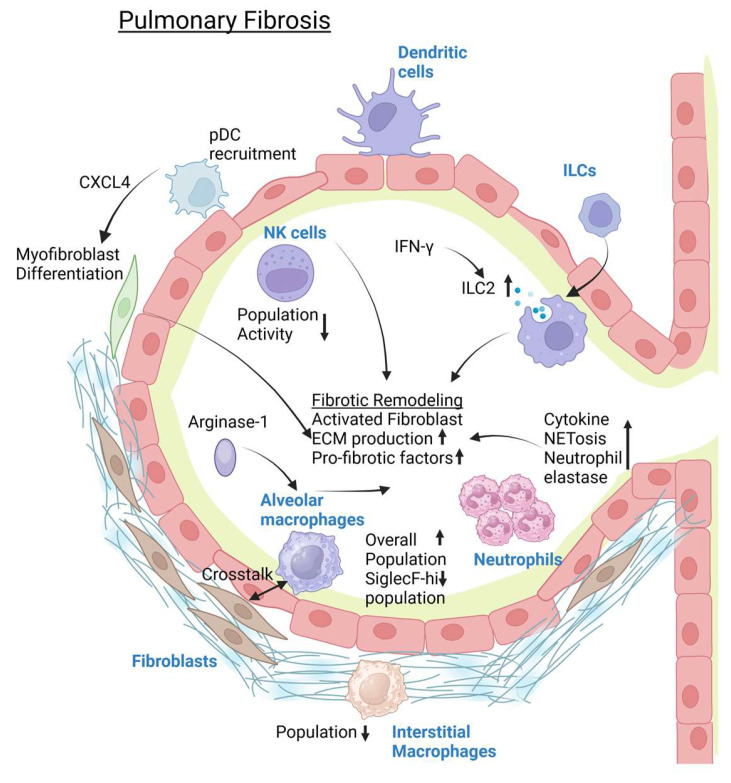
Changes seen in the innate immunity component during pulmonary fibrosis. Created in Biorender. Nagano, T. (2025). https://BioRender.com/y19r764 (accessed on 15 January 2025).

While they are abundant in the lungs and their ability to respond to antigens and pathogens via IL-13 is known, not much has been reported on the role of ILCs in pulmonary fibrosis. ILC2 increase has been identified in the lungs of IPF patients, and its activation via IFN-*γ* signaling reduction has been related to spontaneous pulmonary fibrosis in mice [136]. Moreover, CD-45-deficient mice showed a substantial increase in ILC2s, which leads to a worse fibrotic phenotype [108]. On the other hand, NK cell dysfunction could also affect tissue fibrogenesis, where in IPF patients there is a reduction in the proportion and activity of NK cells [137]. This has been attributed to changes in the microenvironment of the lung. Thus, it is clear that NK cells are important in preventing further remodeling during lung fibrosis. Lastly, the DC population is important in actually promoting fibrosis through its capability to induce myofibroblast differentiation, a major source of collagen and other ECM production [138]. The pDCs are the main culprit for this phenomenon, and this is achieved through the secretion of CXCL4 by pDCs [139]. This is supported in another study, where deletion or inhibition of CXCL4 has been shown to reduce lung fibrosis [140]. CXCL4 can also promote the differentiation of monocytes into pro-inflammatory and pro-fibrotic DCs, potentiating the overt fibrogenesis driven by DCs, highlighting CXCL4 as a potential therapeutic target for lung fibrosis [139]. A schematic figure summarizing the role of innate immune cells in pulmonary fibrosis is shown in Figure 4.

### 3.5. Therapeutic Strategies in Modulating Innate Immunity of the Lung

**Figure 5:**
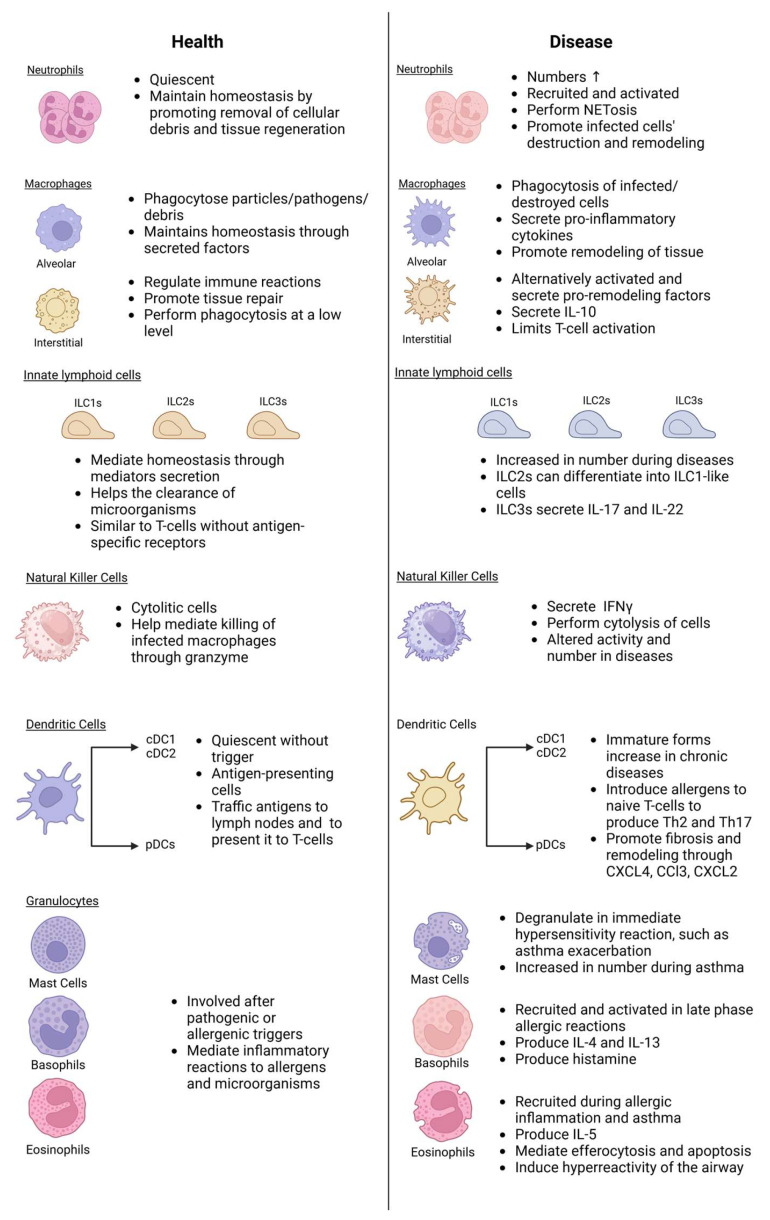
Summary of the changes in innate immune cells during lung diseases. ILC, innate lymphoid cells; cDC, conventional dendritic cells; pDC, plasmacytoid dendritic cells; NET, neutrophil extracellular traps; IL, interleukin; IFN, interferon; Th, T-helper cells; CXCL, C-X-C motif chemokine ligand; CCL, C-C motif chemokine ligand 2. Created in BioRender. Nagano, T. (2025) https://BioRender.com/h88m408 (accessed on 23 December 2024).

As discussed in the previous section, lung diseases are often accompanied by functional and/or molecular changes in the components of the innate immunity system, as also summarized in Figure 5. This opens up the possibility of targeting said alterations to correct the imbalance in the disease-controlling inflammatory process commonly found across lung diseases. First, modulating the acute response of innate immunity through various means has been extensively studied and reviewed. One method to achieve this is by using TLR agonist drugs, to prime the initiation of the inflammatory cascade by TLR activation through PAMPs to properly activate the immune responses and prevent overt infections. This has been mainly investigated pre-clinically in various bacterial (e.g., *P. aeruginosa*) or viral (e.g., influenza) infections [141,142]. Of note, different TLR isoforms are being targeted in different infections, for example, agonists to TLRs 2 and 6, which are not associated with antiviral immunity, are more potent for treating viral infections [141,143].

Targeting the cytokines produced by innate immune cells is another way of controlling excessive inflammation. For instance, anti-TNF-*α* was initially tried in asthma and COPD, but antagonizing TNF-*α* instead caused the occurrence of anti-TNF-*α*-related lung diseases, such as interstitial lung diseases [144,145]. In contrast, the anti-IL-6 agent tocilizumab is rather successful as a drug, and it is widely used for several diseases, such as rheumatoid arthritis or juvenile idiopathic arthritis. In lung diseases, IL-6 has been used for COVID-19 infections and is also approved for systemic sclerosis-associated interstitial lung disease (SSc-ILD). At the same time, it is still being investigated in other conditions such as severe asthma [146,147,148]. Anti-IL-13 agents, such as lebrikizumab or tralokinumab, are also being investigated for asthma, in addition to COPD, due to their involvement in mediating T-cell responses after their release from ILCs and in mediating airway hypersensitivity [149,150]. Besides those mentioned, many different agents targeting various cytokines or other mediators of inflammation are continuously being investigated, such as those targeting the inflammasome NLRP3 [151,152].

Beyond preventing excessive inflammatory processes initiated by innate immunity, optimizing the functions of innate immunity cells is another way to treat lung diseases related to innate immunity. Interestingly, vitamin D has been reported as an important immunomodulator that can help increase the potency of innate immune cells against infections. A meta-analysis of 25 trials reported how vitamin D supplementation could improve protection against acute respiratory infections [153]. Innate immune cells are among the targets for active vitamin D because vitamin D receptors can be found in almost all of the immune cells, including neutrophils, macrophages, and DCs. Vitamin D can induce an increase in chemotaxis and phagocytic ability of macrophages, while it helps DCs induce T-cell polarization to a Th2 phenotype [154]. Vitamin D is also known as an antimicrobial through its capability to induce antimicrobial peptides such as cathelicidins or beta-defensins [154]. Lastly, deficiency in vitamin D not only increases the risk of acute infections but also increases the risk of chronic lung disease occurrence [155,156].

## 4. Conclusions

It is clear that the lung immune system, in particular the innate immune cells discussed in this review, plays a major role in various lung diseases through its capabilities to modulate acute and chronic inflammatory actions in the tissue. Still, many questions remain on the intricacies of the immune cells’ work during different conditions and the interactions between innate immune cells in the lung during pathological conditions. Future studies in this particular field are warranted to progress our understanding regarding the innate immune system in the lung and how we can effectively modulate this system as a therapeutic strategy.

## Data Availability

Data are included in the article.
